# Comparison of Plasma Lipoprotein Composition and Function in Cerebral Amyloid Angiopathy and Alzheimer’s Disease

**DOI:** 10.3390/biomedicines9010072

**Published:** 2021-01-12

**Authors:** Anna Bonaterra-Pastra, Sofia Fernández-de-Retana, Andrea Rivas-Urbina, Núria Puig, Sònia Benítez, Olalla Pancorbo, David Rodríguez-Luna, Francesc Pujadas, Maria del Mar Freijo, Silvia Tur, Maite Martínez-Zabaleta, Pere Cardona Portela, Rocío Vera, Lucia Lebrato-Hernández, Juan F. Arenillas, Soledad Pérez-Sánchez, Joan Montaner, Jose Luis Sánchez-Quesada, Mar Hernández-Guillamon

**Affiliations:** 1 Neurovascular Research Laboratory, Vall d’Hebron Research Institute, Universitat Autònoma de Barcelona, 08035 Barcelona, Spain; annabonaterra95@gmail.com (A.B.-P.); sofia.fernandezderetana@gmail.com (S.F.-d.-R.); montaner.villalonga@gmail.com (J.M.); 2 Cardiovascular Biochemistry Group, Research Institute of the Hospital de Sant Pau (IIB Sant Pau), 08041 Barcelona, Spain; arivas@santpau.cat (A.R.-U.); npuigg@santpau.cat (N.P.); sbenitez@santpau.cat (S.B.); 3 Stroke Research Group, Vall d’Hebron Research Institute, 08035 Barcelona, Spain; olallaprvhir@gmail.com (O.P.); rodriguezluna@vhebron.net (D.R.-L.); 4 Dementia Unit, Neurology Department, Vall d’Hebron Hospital, 08035 Barcelona, Spain; 15078fpn@comb.cat; 5 Neurovascular Group, Biocruces Health Research Institute, 48903 Barakaldo, Spain; MARIADELMAR.FREIJOGUERRERO@osakidetza.net; 6 Department of Neurology, Son Espases University Hospital, 07010 Palma de Mallorca, Spain; silvia.tur@ssib.es; 7 Department of Neurology, Donostia University Hospital, 20014 San Sebastián, Spain; mariateresa.martinezzabaleta@osakidetza.net; 8 Department of Neurology, Bellvitge University Hospital, 08907 L’Hospitalet de Llobregat, Spain; pcardonap@bellvitgehospital.cat; 9 Stroke Unit-Department of Neurology, Ramon y Cajal University Hospital, 28034 Madrid, Spain; rovera78@hotmail.com; 10 Stroke Unit, Virgen del Rocío University Hospital, 41013 Sevilla, Spain; lucia.lebrato.hdez@gmail.com; 11 Stroke Program, Department of Neurology, Hospital Clínico Universitario, 47703 Valladolid, Spain; juanfarenillas@gmail.com; 12 Clinical Neurosciences Research Group, Department of Medicine, University of Valladolid, 47702 Valladolid, Spain; 13 Department of Neurology, Virgen Macarena University Hospital, 41009 Sevilla, Spain; soledad.perez.sanchez@gmail.com; 14 Stroke Research Program, Insitute of Biomedicine of Sevilla, IBiS, 41013 Sevilla, Spain; 15 CIBER de Diabetes y Enfermedades Metabólicas (CIBERDEM), Instituto de Salud Carlos III (ISCIII), 28029 Madrid, Spain

**Keywords:** cerebral amyloid angiopathy, Alzheimer’s disease, lipoprotein composition, lipid profile, apolipoproteins

## Abstract

Cerebral amyloid angiopathy (CAA) refers to beta-amyloid (Aβ) deposition in brain vessels and is clinically the main cause of lobar intracerebral hemorrhage (ICH). Aβ can also accumulate in brain parenchyma forming neuritic plaques in Alzheimer’s disease (AD). Our study aimed to determine whether the peripheral lipid profile and lipoprotein composition are associated with cerebral beta-amyloidosis pathology and may reflect biological differences in AD and CAA. For this purpose, lipid and apolipoproteins levels were analyzed in plasma from 51 ICH-CAA patients (collected during the chronic phase of the disease), 60 AD patients, and 60 control subjects. Lipoproteins (VLDL, LDL, and HDL) were isolated and their composition and pro/antioxidant ability were determined. We observed that alterations in the lipid profile and lipoprotein composition were remarkable in the ICH-CAA group compared to control subjects, whereas the AD group presented no specific alterations compared with controls. ICH-CAA patients presented an atheroprotective profile, which consisted of lower total and LDL cholesterol levels. Plasma from chronic ICH-CAA patients also showed a redistribution of ApoC-III from HDL to VLDL and a higher ApoE/ApoC-III ratio in HDL. Whether these alterations reflect a protective response or have a causative effect on the pathology requires further investigation.

## 1. Introduction

Cerebral beta-amyloidosis is defined as the accumulation of amyloid-beta (Aβ) in the brain and is a principal neuropathological feature in Alzheimer’s disease (AD) and in the most common form of sporadic cerebral amyloid angiopathy (CAA). AD is the most common cause of dementia worldwide whereas CAA is the most frequent cause of lobar intracerebral hemorrhage (ICH) in adults over 55-60 years of age [1,2]. After symptomatic ICH, cognitive deterioration is a relevant clinical manifestation of CAA, independent of AD [3]. CAA is present in nearly all brains with AD [4], although advanced CAA is only present in approximately 25% of AD brains [5]. Even though there is a high overlap between the two diseases, in terms of Aβ level generation and clearance pathways, the pathological mechanisms and clinical presentation differ. While Aβ accumulates in cerebral blood vessels replacing smooth muscle cells and inducing vascular degeneration compromising the vessel functionality and integrity in CAA, in AD Aβ accumulates in brain parenchyma being the core of neuritic plaques contributing to the loss of synapses and neurons [4]. Aβ peptides are caused by the sequential processing of amyloid precursor protein (APP) by β- secretase and then by ɣ-secretase, mainly generating peptides consisting of 40 or 42 amino acids (Aβ40 and Aβ42, respectively). Aβ42 is the main component of amyloid plaques in sporadic AD brains, while Aβ40 is the predominant component in vascular deposits in CAA [6,7]. Actually, Aβ40 levels in cerebrospinal fluid (CSF) have been described to be lower in CAA patients than in AD, whereas Aβ42 is decreased in both [8]. Beyond the Aβ peptide length, the reasons explaining the localization of Aβ peptides in CAA and AD, which involve a different clinical phenotype, are still unidentified. Even though both pathologies present distinctive symptomatology, there are no biomarkers to distinguish them in the clinical practice yet [4]. In this context, cerebral Aβ deposition, parenchymal, and vascular, can be detected by amyloid positron emission tomography (PET) imaging. However, PET’s diagnostic accuracy for CAA is still limited [9].

It is known that tight control of cholesterol is essential for correct brain function [10] and growing evidence shows that cholesterol metabolism in the brain is closely related to the onset of neurocognitive impairment [11]. Cholesterol levels in AD have been extensively studied, and although there are conflicting data [12], a meta-analysis in 2017 reported that high levels of total cholesterol in midlife and early states of aging are significantly associated with a higher risk of developing AD [13]. In this context, lipid-lowering statins have been reported to reduce the risk of AD and decrease its progression [14]. In contrast, it has been demonstrated that low triglycerides, total and LDL cholesterol, and high HDL cholesterol levels, are associated with an increased probability of ICH occurrence, including lobar ICH [15,16,17]. Even though statin treatment as such does not increase the risk of experiencing ICH, it has been suggested that in patients with prior ICH history, this treatment could eventually promote another hemorrhagic event [16,18].

On the other hand, the APOE locus, which encodes ApoE, is the genetic factor most associated with sporadic AD and CAA [19,20,21]. Indeed, the Apoε4 allele is a major risk factor for both AD and CAA and it is associated with neuritic and vascular Aβ deposition [22,23,24]. In contrast, the Apoε2 allele is protective in AD [25] but a risk factor for ICH attributed to CAA [23,26]. This difference regarding the genetic association of the Apoε2 supports a functional involvement of lipid metabolism in the transport and localization of Aβ within the brain, as the involvement of the Apoε2 allele has been related to a major clearance across the blood-brain barrier (BBB) [27]. In addition to the ApoE genotype, polymorphisms in other genes related to lipid metabolism, such as ApoJ, ApoC-III and ApoA-I, have also been reported as genetic determinants of AD risk [28,29,30].

The relationship of lipoproteins with pathological processes is not only related to their concentration but also depends on their qualitative characteristics. Lipoproteins are not homogeneous entities but are formed by multiple heterogeneous particles differing in their relative content of both lipids and proteins [31]. Regarding the protein moiety of lipoproteins, the only common molecule in all very-low-density lipoprotein (VLDL) and low-density lipoprotein (LDL) particles is ApoB-100, whereas ApoA-I is the only protein contained in all the high-density lipoprotein (HDL) particles [32]. However, particularly in HDL, there are a plethora of other apolipoproteins and enzymes with different functions, whose content varies among lipoproteins and confers them in different capacities [33].

In an atherosclerotic disease context, the ability of lipoproteins, mainly HDL and LDL, to enter the arterial wall is well known. As opposed to LDL, which mainly plays a proinflammatory role, the function of HDL includes antioxidant and anti-inflammatory actions protecting the arterial wall from deleterious effects [33]. This function is mediated by apolipoproteins such as ApoA-I or enzymes such as paraoxonase-1 (PON1), lecithin cholesterol acyltransferase (LCAT), or lipoprotein-associated phospholipase A_2_ (Lp-PLA_2_). This implication of lipoproteins in the arterial wall can also have an effect on brain vessel stability in conditions such as CAA. In addition, some apolipoproteins, such as ApoE and ApoJ, are the main transporters of lipids in the brain [10,34], and together with ApoA-I, they can also modulate cerebral Aβ aggregation, deposition, and distribution [27,35,36,37,38,39]. This suggests that alterations in the composition and function of plasma lipoproteins could have a direct role in the formation of amyloid deposits in the arterial wall of brain arteries and/or parenchyma. Based on this assumption, our aim was to analyze the composition and function of lipoproteins isolated from AD or ICH-CAA plasma patients to assess possible abnormalities compared with lipoproteins from sex- and age-matched healthy subjects.

## 2. Experimental Section

### 2.1. Study Population

The population studied in this project consisted of 51 patients who had presented ICH with clinical suspicion of CAA, 60 AD patients, and 60 gender- and age-matched control subjects. The 51 ICH-CAA patients were recruited during a follow-up visit in neurology or stroke units of 10 different Spanish centers. All AD patients and controls were recruited at Vall d’Hebron University Hospital (VHUH).

ICH-CAA patients were >55 years old and had suffered at least one lobar intracerebral hemorrhage. Patients who exhibited any deep intracerebral hemorrhage, presented microbleeds in the basal ganglia, internal or external capsule, thalamus or brainstem, or were being treated with anticoagulant therapy were excluded. The diagnosis was made by magnetic resonance imaging (MRI) acquired following the clinical protocol in each center. In all cases, MRI examinations were obtained using a 1.5-T whole-body scanner. Images obtained included axial T2-weighted turbo spin-echo, axial T1-weighted spin-echo, turbo fluid-attenuated inversion recovery (FLAIR), and axial T2-weighted susceptibility- based echo-planar gradient-echo sequence. All MRI images were evaluated in VHUH by the same neuroradiologist to avoid bias among the different centers. ICH-CAA patients accomplished a CAA diagnosis according to the modified Boston criteria [40] and did not present a diagnosis of dementia at the time of recruitment. According to the modified Boston criteria, 11 patients were classified as possible CAA, 37 probable CAA, and 3 probable CAA with supporting pathology.

The recruited AD patients presented sporadic probable Alzheimer’s disease, according to NIA-AA criteria [41], with mild-to-moderate dementia based on the mini-mental state examination [42]. The MMSE score for the AD cohort was 18 ± 4. AD patients did not present a history of stroke before recruitment. The control subjects were healthy acquaintances or companions of the patients, who were >55 years old and had no history of stroke or dementia (MMSE = 30 ± 0).

The data obtained from the whole cohort included patient coding, inclusion date, demographic characteristics (age, sex), relevant vascular risk factors (HTA, DM, dyslipidemia), and medication, including statin intake. Clinical and anthropometric data of all groups are shown in Table 1. Blood samples of all groups were collected at a follow-up visit. Samples from the ICH-CAA group were obtained at 11 ± 18 months after the last ICH.

The study was approved by the Clinical Investigation Ethical Committee of the Vall d’Hebron University Hospital, Barcelona, Spain (PR(AG)326/2014) and had the approval of the Ethical Committees of all the participating centers. The study was conducted in accordance with the Helsinki Declaration.

Ten mL of blood in EDTA-containing Vacutainer tubes (Becton Dickinson, Franklin Lakes, NJ, USA) was collected from each participant. Blood was centrifuged at 4 °C for 15 min at 2500 rpm and plasma was immediately aliquoted and frozen at −80 °C. APOE genotypes (rs429358 and rs7412) were determined by allelic discrimination using the TaqMan^®^ Genotyping Master Mix (Applied Biosystems, Foster City, CA, USA) and the SNP genotyping mixes C-3084793 and C-904973 (Applied Biosystems) in a Rotor-Gene 6000 Real-Time PCR analyzer (Corbett Life Sciences, Valencia, CA, USA).

### 2.2. Plasma Determinations

The lipid profile, total apolipoproteins, Lp-PLA_2_ activity, LDL size, and HDL subfraction proportion were determined in plasma obtained in EDTA-containing Vacutainer tubes. The lipid profile included total cholesterol, triglycerides, and VLDL, LDL, and HDL cholesterol. The cholesterol in the lipoprotein fractions was routinely quantified using a direct HDL-cholesterol method (HDL-C plus) or by ultracentrifugation when the TG concentration was higher than 3 mmol/L, according to the National Cholesterol Education Program [43]. All these determinations were performed in the Clinical Biochemistry Unit of the VHUH in an AU 5800 autoanalyzer (Beckman Coulter, Pasadena, CA, USA) using reagents from Beckman Coulter. Apolipoproteins B, A-I, A-II, E, and C-III were quantified in the Research Institute of the Hospital de Sant Pau in a Cobas 6000/c501 autoanalyzer using reagents from Roche Diagnostics (ApoB, ApoA-I, Basel, Switzerland) and Kamiya Biomedical Company (ApoA-II, ApoE, ApoC-III, Seattle, WA, USA). ApoJ was determined by ELISA in a subgroup of the cohort (30 controls, 22 ICH-CAA, and 30 AD) (Mabtech, Stockholm, Sweden). The LDL size and HDL subfraction ratio were evaluated by nondenaturing polyacrylamide gradient (2.5–16%) gel electrophoresis (GGE), as described previously [44]. Briefly, the LDL size was measured using a homemade standard containing four bands of LDL, and the HDL2/3 ratio was calculated from the intensity of HDL 2 and HDL 3 bands. Lp-PLA_2_ activity was measured using 2-tio-PAF (Cayman Chemicals, Ann Arbor, MI, USA) as a substrate [45] according to the manufacturer’s instructions. The distribution of Lp-PLA_2_ between lipoprotein fractions was assessed by precipitating ApoB-containing lipoproteins from plasma with dextran sulfate [46].

### 2.3. Lipoprotein Composition

Lipoproteins were isolated by flotation sequential ultracentrifugation according to density: VLDL (1.006–1.019 g/mL), LDL (1.019–1.063 g/mL), and HDL (1.063–1.210 g/mL). Their lipid and apolipoprotein composition was determined by measuring the content of cholesterol, triglycerides, ApoB, ApoA-I (Roche Diagnostics), phospholipids, free cholesterol (Wako Pure Chemical, Osaka, Japan), ApoA-II, ApoE, and ApoC-III (Kamiya Biomedicals, Seattle, WA, USA) in an autoanalyzer Cobas 6000/c501. ApoJ content in isolated lipoproteins was evaluated using commercial ELISA (Mabtech, Stockholm, Sweden). Before ELISA quantification, lipoprotein samples were diluted to the same cholesterol concentration.

### 2.4. LDL and HDL Susceptibility to Oxidation

Lipoproteins were dialyzed against phosphate-buffered saline (PBS) pH 7.4 by gel filtration chromatography in a PD10 column (Sephadex G-25, GE Healthcare, Chicago, IL, USA). Susceptibility to oxidation was evaluated by monitoring the formation of conjugated diene formation at 234 nm in a Synergy HT spectrophotometer (BioTek, Winooski, VT, USA). LDL or HDL at 0.15 mM cholesterol were incubated with 5 μM CuSO_4_, and the lag phase time of the oxidation kinetics was determined [47].

### 2.5. Antioxidant Capacity of HDL

HDL at 0.15 mM cholesterol was incubated with a standard LDL (obtained from a pool of normolipidemic plasma and stored with 10% sucrose at −80 °C), and oxidation was induced by adding 5 μM CuSO_4_. Conjugated diene formation was monitored as described in the previous section. The results are expressed as the capacity of HDL to prolong the lag phase time of the standard LDL alone, as described previously [48].

### 2.6. Statistical Analysis

All the data were analyzed by comparing the three groups: controls, ICH-CAA, and AD patients. The association of categorical variables with the diagnostic groups was studied using contingency tables and a Chi-squared test using the Pearson *p*-value. Significant *p*-values were adjusted by the Bonferroni test when necessary. The distribution of the continuous variables was tested using the Kolmogorov-Smirnov test. If the distribution was normal, one-way ANOVA and Bonferroni’s test for multiple comparisons were performed. If the distribution was not normal, an independent-samples Kruskal-Wallis test with Dunn-Bonferroni adjustment for multiple comparisons was applied. A Forward LR binary logistic regression using the significant variables after multiple comparisons for each study (lipidic profile, each lipoprotein composition, ApoJ distribution, and lipoprotein size), ApoE4 genotype, sex, and age were assessed. The odds ratios (ORs) and 95% confidence intervals (CIs) for the effect on diagnosis were estimated using binary logistic regression analysis. Data are expressed as the mean ± SD for normal distributions or as the median [interquartile range] for non-normal distributions. A *p*-value below 0.05 was considered statistically significant.

## 3. Results

### 3.1. Lipid Profile

All groups presented similar clinical characteristics with a low incidence of diabetes and relatively frequent dyslipidemia and hypertension (Table 1). Statin intake was similar among groups (Ctrl: 12 (21.8%), ICH-CAA: 17 (31.5%), AD: 14 (31.1%), *p*-value: 0.461). Table 2 shows the lipid profile and apolipoprotein concentration in the plasma from the three groups. No significant difference was observed between the control group and AD patients. In contrast, ICH-CAA subjects showed lower levels of ApoA-II than controls, and differences were also found between ICH-CAA and AD patients, with lower levels of total cholesterol and LDL-c in the ICH-CAA group. Although ApoE levels were significantly different when comparing all groups, multiple comparison analysis did not allow reaching statistical significance between cohorts. Both ApoA-II and LDL-c levels remained significantly lower in the ICH-CAA patients after adjustment for the ApoE4 genotype by binary logistic regressions (ApoA-II (dg/L): OR: 0.538 [0.346–0.835], *p* = 0.006; and LDL-c (mg/dL): OR: 1.016 [1.005–1.027], *p* = 0.004) (Supplemental Table S1).

### 3.2. Prevalence of ApoE Genotypes

As expected, the AD group presented a higher incidence of the ApoE4 genotype than the control and ICH-CAA groups (Table 1 and Supplemental Figure S1). No difference in the frequency of the ApoE4 genotype was observed between ICH-CAA patients and control subjects. Regarding the distribution of the ApoE2 genotype, no statistically significant difference was observed among groups (Table 1 and Supplemental Figure S1).

### 3.3. Lipoprotein Composition

VLDL from ICH-CAA patients contained lower cholesterol (total and esterified) levels than those from control subjects and AD patients, and higher ApoC-III levels than those from AD patients (Table 3). These differences in the composition suggest larger VLDL particles in plasma from ICH-CAA patients. No difference was observed between plasma VLDL from AD patients and controls. After adjusting for the ApoE4 genotype, the esterified cholesterol levels remained significantly different between the ICH-CAA group and control (OR: 0.808 [0.683–0.956], *p* = 0.013); and ApoCIII levels between ICH-CAA group and AD (OR: 0.39 [0.184–0.829], *p* = 0.014) (Supplemental Table S2).

No difference in LDL composition was observed among the studied groups (Table 4). Only a trend toward lower esterified cholesterol content in the ICH-CAA cohort compared to the control and AD groups was detected.

Regarding the composition of HDLs, total and esterified cholesterol levels were increased in plasma from the ICH-CAA group compared with that from both the control and AD groups (Table 5), which is suggestive of the presence of more mature HDL particles in this group. Additionally, the ApoE level showed a trend to increase, whereas ApoC-III was decreased in ICH-CAA patients compared with controls and AD patients, respectively, resulting in a higher ApoE/ApoC-III ratio in the ICH-CAA cohort compared to controls. No difference was observed between AD patients and controls. After adjusting for the ApoE4 genotype, esterified cholesterol levels remained significantly different between ICH-CAA and both controls (OR: 1.583 [1.119–2.239], *p* = 0.010) and AD (OR: 0.525 [0.365–0.754], *p* = 0.0005), and ApoC-III levels also remained significantly different between ICH-CAA and controls (OR: 0.414 [0.221–0.772], *p* = 0.006) (Supplemental Table S3).

### 3.4. Apolipoprotein J Content in Lipoproteins

Levels of ApoJ were also quantified in a subgroup of patients with the aim of studying whether the distribution of this apolipoprotein among the different lipoproteins differed between the selected clinical groups. However, the results showed that the ApoJ content in each lipoprotein fraction was similar between groups (Table 6). A tendency toward higher ApoJ levels in LDL was found in AD patients when the three groups were analyzed. Indeed, ApoJ levels in LDL from AD patients were significantly higher than the corresponding levels in plasma from ICH-CAA patients when adjusted for the ApoE4 genotype (OR: 1.015 [1.002–1.027], *p*= 0.019) (Supplemental Table S4).

### 3.5. Lipoprotein Size and Oxidation-Related Functions

GGE allows us to define the LDL subfraction phenotype of patients. LDL subfraction phenotype A (large LDL particles > 25.5 nm) is the usual phenotype present in normolipidemic healthy subjects, in contrast to phenotype B (small LDL particles < 25.5 nm), which is characteristic of hypertriglyceridemic subjects at high cardiovascular risk. GGE showed that LDL particles from AD patients were slightly smaller than LDL particles from the ICH-CAA group (Table 7), reflecting the differences in lipid profiles between the two groups. However, in all groups, including AD patients, the LDL subfraction phenotype was type A; therefore, it can be considered non-atherogenic. No differences in the HDL2/HDL3 ratio were observed among groups. Regarding Lp-PLA activity, in plasma from AD patients, a decrease in the relative proportion of this activity associated with HDL was observed. No difference was detected in either LDL or HDL susceptibility to oxidation or in the antioxidant capacity of HDL among groups. After adjusting for the ApoE4 genotype, both the LDL size and Lp-PLA relative activity remained significantly lower in the AD group when compared to IHC-CAA (OR: 0.55 [0.331–0.912], *p* = 0.021; OR: 0.962 [0.925–1], *p* = 0.049 (Supplemental Table S5).

## 4. Discussion

This study was conducted to determine whether a complete peripheral lipid analysis in plasma can reveal functional or quantitative circulating markers associated with cerebral beta-amyloidosis pathology and/or reflect biological differences in AD and CAA patients.

Our results showed that alterations in the lipid profile and lipoprotein composition were more evident in ICH-CAA than in AD patients compared to sex- and age-matched control subjects. We found that the lipid profile of the AD cohort was very similar to that of the control subjects. In contrast, patients in a chronic phase after an ICH associated with CAA presented multiple differences, in both the basic lipid profile and lipoprotein composition, compared with control and AD subjects. Even though statin treatment can modify the lipid profile, no relationship was found between statin intake and diagnosis groups, implying that statin treatment did not have an important effect on the obtained results. In addition, the ApoE genotype frequency in our cohort was similar between controls and ICH-CAA patients; hence, differences found in the lipid profile of ICH-CAA patients cannot be attributed to the ApoE genotype either. From a cardiovascular risk point of view, the lipid profile of ICH-CAA subjects was rather atheroprotective, displaying lower levels of total and LDL cholesterol. Other potential cardiovascular risk factors, such as hypertension and diabetes, were similar among groups, and smoking or sedentarism were not studied. This result agrees with previous findings reporting an association between total and LDL cholesterol and increased risk of ICH [12,15,16]. Actually, a prior study reported that a decline in total and LDL cholesterol occurs within 6 months preceding ICH [49]. Indeed, the ApoE2 genotype, which is a risk factor for ICH in CAA, is also associated with lower levels of LDL cholesterol [50]. Lower LDL cholesterol levels were described to be associated with hematoma growth and increased mortality after acute ICH for both lobar and deep localizations [51]. In addition, previous evidence showed that lower total cholesterol and higher HDL cholesterol levels were associated with the presence of specifically lobar cerebral microbleeds [52,53], a characteristic trait of CAA [54]. Since lipids are an essential complement of cellular membranes, it has been proposed that lower blood cholesterol levels and triglycerides may cause fragility and necrosis in endothelial and smooth muscle cells in arterial media, contributing to vessel rupture in patients with ICH or presenting with multiple CMB [16,55,56]. However, the exact pathological mechanisms are still unclear, and other possible explanations, such as the effect of cholesterol on platelet aggregability, are also plausible [51]. On the other hand, our data show that ApoA-II plasma levels were exclusively decreased in the ICH-CAA group, whereas the other apolipoproteins presented similar concentrations among groups. Only a few reports have studied ApoA-II in subjects with cognitive impairment, and the results are divergent. Whereas Song et al. reported that a low concentration of ApoA-II was associated with an increased risk for cognitive decline in normal individuals [57], Lehallier et al. described increased ApoA-II levels in patients with the progression of mild cognitive impairment to AD [58]. Further studies are necessary to confirm or refute whether ApoA-II levels in the blood are associated with the development or progression of CAA.

Regarding the lipoprotein composition, alterations in the lipid content of VLDL in ICH-CAA suggest larger particles. The mechanism leading to this higher size of VLDL is probably related to the increased ApoC-III content since this apolipoprotein is the main inhibitor of the enzyme lipoprotein lipase (LpL), which degrades VLDL triglycerides in the capillary endothelium [59]. Interestingly, increased ApoC-III in VLDL was accompanied by decreased ApoC-III bound to HDL, which may reflect a redistribution of this apolipoprotein among plasma lipoproteins in ICH-CAA patients. In addition, HDL particles from these patients presented a higher ApoE/ApoC-III ratio than those from controls and AD patients and, thus, being a potential biomarker for ICH-CAA. Besides, our data suggest that HDL from ICH-CAA are more mature particles with increased esterified cholesterol. Hence, from a cardiovascular point of view, HDL from ICH-CAA subjects, with more ApoE and less ApoC-III, would also be atheroprotective by enhancing reverse cholesterol transport [60,61,62], in accordance with the atheroprotective lipid profile found in plasma from ICH-CAA patients. Moreover, these data open the possibility that the alterations observed in ICH-CAA HDL particles, especially the increase in ApoE in relation to a decreased ApoC-III, could be a defensive response against the deposition of Aβ in the walls of the brain vasculature, which would be in line with recent results showing that ApoE-enriched HDL reduces CAA in an in vitro model [63].

Concerning lipoprotein composition in the AD cohort, ApoC-III levels in HDL also tended to be lower than in controls. Indeed, higher ApoC-III levels in HDL have been associated with lower dementia and AD risk [64]. We also found low content of Lp-PLA_2_ activity in HDL from AD patients, which could suggest impaired anti-inflammatory function. The physiological implications of such observation in the context of AD are difficult to appraise. However, the antioxidative and anti-inflammatory enzymes transported by HDL play a key role in the maintenance of a systemic non-inflammatory status in blood. Therefore, this finding could be related to the concept that inflammation is a central mechanism in the development of AD [65], which would be reflected at a systemic level in decreased anti-inflammatory capacity of HDL.

We were particularly interested in studying the distribution of ApoJ in circulating lipoproteins because of its potential to participate in Aβ accumulation and clearance and modulate the balance between Aβ levels in brain vessels and parenchymal plaques [66]. In addition, ApoJ is co-deposited with fibrillary Aβ in both parenchymal plaques and vascular Aβ deposits [67,68,69,70]. Therefore, we first analyzed the levels of circulating total ApoJ levels, although no statistically significant differences among groups were obtained. It is worth mentioning that previous studies have found increased ApoJ levels in the plasma of AD and ICH-CAA patients [71,72,73], but other studies have not confirmed such differences [64,74]. However, it has been proposed that ApoJ levels are associated with AD in an age-dependent manner, especially in individuals above 80 years old, as a protective response to brain injury [75], which could explain our results in a slightly younger cohort. Furthermore, ApoJ is a chaperone that interacts with Aβ and prevents its fibrillation and toxicity in vitro [36,76,77] and it is also involved in the clearance of Aβ across the BBB [37,38]. In this sense, we observed higher ApoJ levels in LDL from AD patients, which suggests a redistribution of ApoJ in lipoproteins in AD. Since it has been previously demonstrated that lipidated ApoJ has a major affinity for one of the receptors involved in Aβ clearance through the BBB (LRP-2) [78], the increase in ApoJ levels in LDL could be seen as a protective response to enhance parenchymal Aβ clearance. However, the meaning of our findings regarding abnormal ApoJ distribution in lipoproteins and their link to AD pathology warrants further research and confirmation in other cohorts.

The results obtained in this study do not allow us to consider these lipid-related variables as biomarkers for diagnosis or clinical follow-up in medical practice. Nonetheless, our data can provide some insights to elucidate the relevance of lipid metabolism in the cerebral amyloidosis process and cerebral vascular functionality, which could potentially help in the management and treatment of these diseases. In future studies, it would be interesting to evaluate plasma Aβ levels in the different lipoproteins and thereby extend our results. Analyzing the Aβ distribution among circulating lipoproteins could also deepen our understanding of the role and function of lipoproteins in cerebral beta-amyloidosis. Unfortunately, the Aβ levels in lipoproteins were too low to be detected in the samples used in this study.

The biological overlap between CAA and AD pathologies involves an intrinsic limitation to differentiate the cohorts of the study. Although AD patients and controls did not present a history of ICH, the lack of neuroimaging data in those cohorts could have masked the presence of CAA-related radiological markers. To overcome this limitation and define a clear CAA phenotype in comparison to other potential degrees of CAA pathology, we selected only patients with at least one lobar ICH (ICH-CAA cohort) and without microbleeds in deep cerebral structures nor diagnosis of dementia. Nevertheless, even if there were some common pathological features in the AD and ICH-CAA cohorts, it would not determine the clear and specific lipid profile signature found in the last group.

In addition, the comparison of the three cohorts of the study suggests the analysis of plasma biomarkers of patients who have suffered a symptomatic intracerebral hemorrhage with patients without stroke. We tried to overcome this limitation by selecting plasma samples from ICH-CAA patients in a chronic phase of the disease, avoiding changes in biomarkers due to the inflammatory process caused during the acute phase of the stroke.

It is also important to keep in mind that the data obtained in the ICH-CAA cohort may reflect pathological changes associated with the previous symptomatic hemorrhage within the brain rather than the deposition of Aβ along the cerebral vasculature. In fact, a similar lipid profile has been previously found in non-lobar ICH patients before and immediately after the hemorrhagic episode [15,16,49]. Therefore, lipid analysis of patients who have suffered an ICH independent of a CAA etiology in a chronic phase of the disease would serve to compare and reinterpret our results. Furthermore, our study presents other limitations. First, the sample size is small, which could be a cause of patient selection bias. Larger studies with a higher number of patients should be conducted to confirm our results. Second, the observational nature of our study does not allow us to discriminate whether the differences observed between groups are a cause, a consequence, or a response to the pathology.

## 5. Conclusions

Our data show that, unlike AD, the lipid profile and lipoprotein composition in samples from chronic ICH-CAA patients present numerous differences from those in control subjects. Specifically, we observed an atheroprotective profile associated with ICH-CAA diagnosis, which confirmed previous studies and consisted of lower blood total and LDL cholesterol levels. In this study, ICH-CAA patients also presented a redistribution of ApoC-III from HDL to VLDL and a higher ApoE/ApoC-III ratio in HDL. Whether the alterations observed in lipoproteins from the ICH-CAA cohort are a reflection of a protective response or have a causative effect requires further investigation.

## Figures and Tables

**Table 1:** Demographic and clinical characteristics.

Parameters	Control	ICH-CAA	AD	*p*-Value
Age	76.0 [71.0–81.0]	77.0 [72.0–79.0]	77.0 [72.3–81.0]	0.755
Gender (F)	34 (56.7%)	32 (62.7%)	42 (70%)	0.317
Hypertension	34 (63.0%)	22 (43.1%)	27 (61.4%)	0.083
Diabetes	7 (13.0%)	5 (9.8%)	7 (15.9%)	0.672
Dyslipidemia	19 (35.2%)	17 (34.7%)	22 (51.2%)	0.189
ApoE2	7 (11.7%)	6 (11.8%)	2 (3.3%)	0.181
ApoE4	10 (16.7%)	11 (21.6%)	28 (46.7%) ^$,^**	**0.001**

Age is expressed as median [interquartile range]. ** *p* < 0.001 vs. the control group; ^$^ *p* < 0.05 vs. the ICH-CAA group. Bold numbers indicate statistically significant differences.

**Table 2:** Lipid profile and apolipoprotein concentration in plasma.

Parameters	Controls	ICH-CAA	AD	*p*-Value
Total cholesterol	228.00 [199.50–261.50]	207.00 [185.00–226.00] *	230.00 [198.00–283.00] ^$^	**0.006**
HDL-c	58.30 ± 11.32	58.57 ± 13.09	60.52 ± 15.98	0.642
LDL-c	145.00 [108.00–172.20]	126.00 [114.00–139.60]	143.70 [117.90–190.65] ^$^	**0.004**
VLDL-c	24.80 [18.00–32.00]	21.00 [16.60–26.80]	21.50 [17.50–29.00]	0.150
Triglycerides	122.00 [91.50–162.50]	105.00 [83.00–134.00]	108.50 [87.00–145.00]	0.133
ApoA-I	1.744 ± 0.266	1.707 ± 0.329	1.670 ± 0.284	0.399
ApoA-II	0.416 [0.337–0.460]	0.340 [0.260–0.423] *	0.400 [0.340–0.440]	**0.010**
ApoB	1.095 [0.938–1.285]	0.995 [0.895–1.095]	1.070 [0.900–1.300]	0.052
ApoC-III	0.126 ± 0.054	0.116 ± 0.045	0.107 ± 0.049	0.112
ApoE	0.053 ± 0.016	0.045 ± 0.018	0.053 ± 0.016	**0.030**
ApoJ	0.218 ± 0.059	0.193 ± 0.060	0.211 ± 0.054	0.079

Lipids are expressed as mg/dL and apolipoproteins are expressed as g/L. * *p* < 0.05 vs. the control group; ^$^ *p* < 0.05 vs. the ICH-CAA group.

**Table 3:** VLDL composition.

Parameters	Controls	ICH-CAA	AD	*p*-Value
Cholesterol	20.15 [18.13–21.78]	17.70 [16.60–20.10] *	20.65 [18.00–22.70] ^$^	**0.001**
Free cholesterol	6.65 [6.03–7.45]	6.30 [5.90–6.70]	6.80 [6.10–7.50] ^$^	**0.031**
Esterified cholesterol	13.36 ± 1.90	12.12 ± 3.03 *	13.62 ± 2.92 ^$^	**0.008**
Triglycerides	49.66 ± 4.79	50.73 ± 5.52	48.23 ± 6.07	0.056
Phospholipid	19.70 [18.33–20.48]	19.10 [18.30–20.20]	19.40 [18.53–20.70]	0.731
Protein	11.23 [10.59–11.90]	10.93 [9.87–12.10]	11.44 [9.89–12.75]	0.750
ApoB-100	10.07 [9.30–10.79]	9.60 [8.02–10.08]	10.20 [8.48–12.10]	0.149
ApoC-III	0.70 [0.50–1.00]	0.90 [0.40–1.30]	0.60 [0.23–0.85] ^$^	**0.022**
ApoE	0.40 [0.13–0.68]	0.47 [0.20–0.80]	0.36 [0.17–0.68]	0.724

Data are expressed as the percentage of each component of the total lipoprotein mass. * *p* < 0.05 vs. the control group; ^$^ *p* < 0.05 vs. the ICH-CAA group.

**Table 4:** LDL composition.

Parameters	Controls	ICH-CAA	AD	*p*-Value
Cholesterol	39.20 [38.10–40.30]	38.60 [36.50–40.40]	39.30 [38.10–40.25]	0.288
Free cholesterol	10.30 [9.70–10.68]	10.40 [9.80–10.80]	10.30 [9.65–10.68]	0.755
Esterified cholesterol	29.10 [28.28–29.88]	28.20 [26.70–30.00]	29.15 [28.13–29.90]	0.062
Triglycerides	7.65 [6.90–8.88]	8.40 [6.90–9.60]	7.70 [6.70–9.00]	0.334
Phospholipid	25.40 [24.70–25.98]	25.40 [24.88–26.00]	25.50 [24.80–26.00]	0.766
Protein	27.50 [26.95–28.38]	27.40 [26.60–28.40]	27.37 [26.76–28.24]	0.587
ApoB-100	27.37 [26.73–28.28]	27.43 [26.45–28.40]	27.24 [26.60–28.13]	0.723
ApoE	0.10 [0.05–0.20]	0.14 [0.09–0.21]	0.12 [0.06–0.18]	0.294

Data are expressed as the percentage of each component of the total lipoprotein mass.

**Table 5:** HDL composition.

Parameters	Controls	ICH-CAA	AD	*p*-Value
Cholesterol	16.04 ± 1.61	17.06 ± 1.70 *	16.32 ± 1.42 ^$^	**0.003**
Free cholesterol	3.10 [2.80–3.48]	3.30 [2.80–3.70]	3.20 [2.90–3.60]	0.487
Esterified cholesterol	12.92 ± 1.42	13.86 ± 1.30 *	13.08 ± 1.13 ^$^	**<0.001**
Triglycerides	3.05 [2.70–3.80]	3.00 [2.00–4.00]	3.20 [2.53–3.90]	0.444
Phospholipid	28.67 ± 2.63	28.68 ± 2.36	29.06 ± 1.76	0.567
Protein	51.92 ± 2.82	51.08 ± 2.80	51.34 ± 2.17	0.214
ApoA-I	38.98 ± 3.10	39.11 ± 2.70	38.94 ± 2.65	0.948
ApoA-II	10.97 ± 1.86	10.44 ± 1.81	10.68 ± 1.87	0.318
ApoC-III	1.59 [0.93–2.00]	0.95 [0.60–1.35] *	0.98 [0.64–1.79]	**0.003**
ApoE	0.38 [0.21–0.50]	0.47 [0.26–0.80]	0.36 [0.21–0.46]	0.081
ApoE/ApoC-III	0.25 [0.13–0.39]	0.50 [0.25–0.92] *	0.28 [0.13–0.65]	**0.007**

Data are expressed as the percentage of each component of the total lipoprotein mass. * *p* < 0.05 vs. the control group; ^$^ *p* < 0.05 vs. the ICH-CAA group.

**Table 6:** ApoJ content in lipoproteins.

Parameters	Controls	ICH-CAA	AD	*p*-Value
ApoJ in HDL	988.07 ± 405.68	1106.81 ± 349.20	1000.17 ± 270.93	0.430
ApoJ LDL	97.46 [73.84–189.11]	104.27 [81.81–144.17]	146.54 [97.46–221.94]	0.064
ApoJ VLDL	218.64 [170.18–309.78]	249.60 [167.51 –318.45]	223.08 [178.98–332.63]	0.782

Data are expressed as µg apoJ/mmol cholesterol.

**Table 7:** Lipoprotein size, Lp-PLA_2_ activity and oxidative properties of LDL and HDL.

Parameters	Controls	ICH-CAA	AD	*p*-Value
LDL size (nm)	26.23 [25.83–26.89]	26.40 [25.90–26.86]	26.13 [25.52–26.50] ^$^	**0.020**
Ratio HDL2/HDL3	0.86 [0.38–1.23]	0.96 [0.56–1.44]	0.91 [0.45–1.37]	0.611
Total Lp-PLA_2_ activity ^1^	18.30 [15.40–22.55]	17.30 [14.17–21.80]	18.32 [16.10–21.45]	0.415
Lp-PLA_2_ activity in HDL ^1^	7.85 [6.53–9.80]	8.18 [7.30–9.73]	7.75 [6.80–8.63]	0.381
Lp-PLA_2_ activity in HDL (%)	43.16 [36.97–52.18]	46.49 [42.09–54.55]	40.25 [35.83–48.24] ^$^	**0.029**
HDL lag time (min)	25.04 ± 5.88	27.09 ± 4.85	25.71 ± 5.66	0.165
LDL lag time (min)	46.00 [38.71–51.93]	46.00 [38.50–50.60]	46.20 [40.18–5.45]	0.904
Antioxidant ability of HDL ^2^	121.48 ± 47.21	124.02 ± 50.73	105.95 ± 44.31	0.104

^1^ µmol/min mL. ^2^ percentage of increase in lag time. ^$^ *p* < 0.05 vs. the ICH-CAA group.

## Data Availability

The data presented in this study are available on reasonable request from the corresponding author.

## Supplementary Materials

The following are available online at https://www.mdpi.com/2227-9059/9/1/72/s1. Table S1. Binary logistic regression for lipid profile and apolipoprotein concentration in plasma. Table S2. Binary logistic regression for VLDL composition. Table S3. Binary logistic regression for HDL composition. Table S4. Binary logistic regression for ApoJ content in lipoproteins. Table S5. Binary logistic regression for lipoprotein size. Figure S1. ApoE genotype.

## References

[1] Vinters H.V. (2015). Emerging Concepts in Alzheimer’s Disease. Annu. Rev. Pathol. Mech. Dis..

[2] Charidimou A., Boulouis G., Gurol M.E., Ayata C., Bacskai B.J., Frosch M.P., Viswanathan A., Greenberg S.M. (2017). Emerging concepts in sporadic cerebral amyloid angiopathy. Brain.

[3] Arvanitakis Z., Leurgans S.E., Wang Z., Wilson R.S., Bennett D.A., Schneider J.A. (2011). Cerebral Amyloid Angiopathy Pathology and Cognitive Domains in Older Persons. Ann. Neurol..

[4] Greenberg S.M., Bacskai B.J., Hernandez-Guillamon M., Pruzin J., Sperling R., van Veluw S.J. (2020). Cerebral amyloid angiopathy and Alzheimer disease—One peptide, two pathways. Nat. Rev. Neurol..

[5] Ellis R.J., Olichney J.M., Thal L.J., Mirra S.S., Morris J.C., Beekly D., Heyman A. (1996). Cerebral amyloid angiopathy in the brains of patients with Alzheimer’s disease: The CERAD experience, Part XV. Neurology.

[6] Suzuki N., Iwatsubo T., Odaka A., Ishibashi Y., Kitada C., Ihara Y. (1994). High tissue content of soluble beta 1-40 is linked to cerebral amyloid angiopathy. Am. J. Pathol..

[7] Yamada M. (2015). Cerebral amyloid angiopathy: Emerging concepts. J. Stroke.

[8] Renard D., Castelnovo G., Wacongne A., Le Floch A., Thouvenot E., Mas J., Gabelle A., Labauge P., Lehmann S. (2012). Interest of CSF biomarker analysis in possible cerebral amyloid angiopathy cases defined by the modified Boston criteria. J. Neurol..

[9] Banerjee G., Carare R., Cordonnier C., Greenberg S.M., Schneider J.A., Smith E.E., Van Buchem M., Van Der Grond J., Verbeek M.M., Werring D.J. (2017). The increasing impact of cerebral amyloid angiopathy: Essential new insights for clinical practice. J. Neurol. Neurosurg. Psychiatry.

[10] Wang H., Eckel R.H. (2014). What are Lipoproteins doing in the Brain?. Trends Endocrinol Metab..

[11] Loera-Valencia R., Goikolea J., Parrado-Fernandez C., Merino-Serrais P., Maioli S. (2019). Alterations in cholesterol metabolism as a risk factor for developing Alzheimer’s disease: Potential novel targets for treatment. J. Steroid Biochem. Mol. Biol..

[12] Appleton J.P., Scutt P., Sprigg N., Bath P.M. (2017). Hypercholesterolaemia and vascular dementia. Clin. Sci..

[13] Anstey K.J., Ashby-mitchell K., Peters R. (2017). Updating the Evidence on the Association between Serum Cholesterol and Risk of Late-Life Dementia: Review and Meta-Analysis. J. Alzheimer’s Dis..

[14] Chu C.S., Tseng P.T., Stubbs B., Chen T.Y., Tang C.H., Li D.J., Yang W.C., Chen Y.W., Wu C.K., Veronese N. (2018). Use of statins and the risk of dementia and mild cognitive impairment: A systematic review and meta-analysis. Sci. Rep..

[15] Wang X., Dong Y., Qi X., Huang C., Hou L. (2013). Cholesterol levels and risk of hemorrhagic stroke: A systematic review and meta-analysis. Stroke.

[16] Ma Y., Li Z., Chen L., Li X. (2016). Blood lipid levels, statin therapy and the risk of intracerebral hemorrhage. Lipids Health Dis..

[17] Pezzini A., Grassi M., Iacoviello L., Zedde M., Marcheselli S., Silvestrelli G., DeLodovici M.L., Sessa M., Zini A., Paciaroni M. (2016). Serum cholesterol levels, HMG-CoA reductase inhibitors and the risk of intracerebral haemorrhage. The Multicenter Study on Cerebral Haemorrhage in Italy (MUCH-Italy). J. Neurol. Neurosurg. Psychiatry.

[18] Endres M., Nolte C.H., Scheitz J.F. (2018). Statin treatment in patients with intracerebral hemorrhage. Stroke.

[19] Schmechel D.E., Saunders A.M., Strittmatter W.J., Crain B.J., Hulette C.M., Joo S.H., Pericak-Vance M.A., Goldgaber D., Roses A.D. (1993). Increased amyloid β-peptide deposition in cerebral cortex as a consequence of apolipoprotein E genotype in late-onset Alzheimer disease. Proc. Natl. Acad. Sci. USA.

[20] Greenberg S.M., Rebeck G.W., Vonsattel J.P.G., Gomez-isla T., Hyman B.T. (1995). Apolipoprotein E ε4 and Cerebral Hemorrhage Associated with Amyloid Angopathy. Ann. Neurol..

[21] Marais A.D. (2019). Apolipoprotein E in lipoprotein metabolism, health and cardiovascular disease. Pathology.

[22] Olichney J.M., Hansen L.A., Galasko D., Saitoh T., Hofstetter C.R., Katzman R., Thal L.J. (1996). The apolipoprotein E epsilon 4 allele is associated with increased neuritic plaques and cerebral amyloid angiopathy in Alzheimer’s disease and Lewy body variant. Neurology.

[23] Nicoll J.A., McCarron M.O. (2001). APOE gene polymorphism as a risk factor for cerebral amyloid angiopathy-related hemorrhage. Amyloid.

[24] Liu Y., Wang H., Han P.-R., Tan C., Wang C., Meng X.-F., Risacher S.L., Saykin A.J. (2015). APOE genotype and neuroimaging markers of Alzheimer’s disease: Systematic review and meta-analysis. J. Neurol. Neurosurg. Psychiatry.

[25] Corder E.H., Saunders A.M., Risch N.J., Strittmatter W.J., Schmechel D.E., Gaskell P.C., Rimmler J.B., Locke P.A., Conneally P.M., Schmader K.E. (1994). Protective effect of apolipoprotein E type 2 allele for late onset Alzheimer disease. Nat. Genet..

[26] Greenberg S.M., Vonsattel J.-P.G., Segal A.Z., Chiu R.I., Clatworthy A.E., Liao A., Hyman B.T., Rebeck G.W. (1998). Association of apolipoprotein E and ε2 vasculopathy in cerebral amyloid angiopathy. Neurology.

[27] Deane R., Sagare A., Hamm K., Parisi M., Lane S., Finn M.B., Holtzman D.M., Zlokovic B.V. (2008). ApoE isoform-specific disruption of amyloid β peptide clearance from mouse brain. J. Clin. Investig..

[28] Foster E.M., Dangla-Valls A., Lovestone S., Ribe E.M., Buckley N.J. (2019). Clusterin in Alzheimer’s disease: Mechanisms, genetics, and lessons from other pathologies. Front. Neurosci..

[29] Sun Y., Shi J., Zhang S., Tang M., Han H., Guo Y., Ma C., Liu X., Li T. (2005). The APOC3 SstI polymorphism is weakly associated with sporadic Alzheimer’s disease in a Chinese population. Neurosci. Lett..

[30] Vollbach H., Heun R., Morris C.M., Edwardson J.A., McKeith I.G., Jessen F., Schulz A., Maier W., Kölsch H. (2005). APOA1 polymorphism influences risk for early-onset nonfamiliar AD. Ann. Neurol..

[31] Hoofnagle A.N., Heinecke J.W. (2009). Lipoproteomics: Using mass spectrometry-based proteomics to explore the assembly, structure, and function of lipoproteins. J. Lipid Res..

[32] Alaupovic P. (1991). Apolipoprotein composition as the basis for classifying plasma lipoproteins. Characterization of ApoA- and ApoB-containing lipoprotein families. Prog. Lipid Res..

[33] Kontush A., Chapman M.J. (2010). Antiatherogenic function of HDL particle subpopulations: Focus on antioxidative activities. Curr. Opin. Lipidol..

[34] Stukas S., Robert J., Wellington C.L. (2014). High-density lipoproteins and cerebrovascular integrity in Alzheimer’s disease. Cell Metab..

[35] Huynh T.P.V., Davis A.A., Ulrich J.D., Holtzman D.M. (2017). Apolipoprotein E and Alzheimer’s disease: The influence of apolipoprotein E on amyloid-β and other amyloidogenic proteins. J. Lipid Res..

[36] Narayan P., Orte A., Clarke R.W., Bolognesi B., Ganzinger K.A., Meehan S., Wilson M.R., Christopher M. (2016). The extracellular chaperone clusterin sequesters oligomeric forms of the Aβ1–40 peptide. Nat. Struct. Mol. Biol..

[37] Bell R.D., Sagare A., Friedman A.E., Bedi G., Holtzman D.M., Deane R., Zlokovic B.V. (2007). Transport pathways for clearance of human Alzheimer’s amyloid β-peptide and apolipoproteins E and J in the mouse central nervous system. J. Cereb. Blood Flow Metab..

[38] Merino-Zamorano C., De Retana S.F., Montañola A., Batlle A., Saint-Pol J., Mysiorek C., Gosselet F., Montaner J., Hernández-Guillamon M. (2016). Modulation of Amyloid-β1-40 Transport by ApoA1 and ApoJ Across an in vitro Model of the Blood-Brain Barrier. J. Alzheimer’s Dis..

[39] Hottman D.A., Chernick D., Cheng S., Wang Z., Li L. (2014). HDL and Cognition in Neurodegenerative Disorders. Neurobiol Dis..

[40] Linn J., Halpin A., Demaerel P., Ruhland J., Giese A., Dichgans M., van Buchem M.B.H. (2010). Prevalence of Superficial Siderosis in Patients with Cerebral Amyloid Angiopathy. Neurology.

[41] McKhann G., Knopman D.S., Chertkow H., Hyman B.T., Jack C.R., Kawas C.H., Klunk W.E., Koroshetz W.J., Manly J.J., Mayeux R. (2011). the diagnosis of dementia due to Alzheimer’s disease: Recommendations from the National Institute on Aging-Alzheimer’s Association workgroups on diagnostic guidelines for Alzheimer’s disease. Alzheimers Dement..

[42] Folstein M.F., Folstein S.E., McHugh P.R. (1975). “Mini-mental state”: A practical method for grading the cognitive state of patients for the clinician. J. Psychiatr. Res..

[43] NCEP (2002). Third Report of the National Cholesterol Education Program (NCEP) Expert Panel on Detection, Evaluation, and Treatment of High Blood Cholesterol in Adults (Adult Treatment Panel III) final report. Circulation.

[44] Sánchez-Quesada J.L., Benítez S., Otal C., Franco M., Blanco-Vaca F., Ordóñez-Llanos J. (2002). Density distribution of electronegative LDL in normolipemic and hyperlipemic subjects. J. Lipid Res..

[45] Benítez S., Sánchez-Quesada J.L., Ribas V., Jorba O., Blanco-Vaca F., González-Sastre F., Ordóñez-Llanos J. (2003). Platelet-activating factor acetylhydrolase is mainly associated with electronegative low-density lipoprotein subfraction. Circulation.

[46] Sánchez-Quesada J.L., Vinagre I., De Juan-Franco E., Sánchez-Hernández J., Bonet-Marques R., Blanco-Vaca F., Ordóñez-Llanos J., Pérez A. (2013). Impact of the LDL subfraction phenotype on Lp-PLA2 distribution, LDL modification and HDL composition in type 2 diabetes. Cardiovasc. Diabetol..

[47] Benítez S., Sánchez-Quesada J.L., Lucero L., Arcelus R., Ribas V., Jorba O., Castellví A., Alonso E., Blanco-Vaca F., Ordóñez-Llanos J. (2002). Changes in low-density lipoprotein electronegativity and oxidizability after aerobic exercise are related to the increase in associated non-esterified fatty acids. Atherosclerosis.

[48] De Juan-Franco E., Pérez A., Ribas V., Sánchez-Hernández J.A., Blanco-Vaca F., Ordóñez-Llanos J., Sánchez-Quesada J.L. (2009). Standardization of a method to evaluate the antioxidant capacity of high-density lipoproteins. Int. J. Biomed. Sci..

[49] Phuah C.L., Raffeld M.R., Ayres A.M., Viswanathan A., Greenberg S.M., Biffi A., Rosand J., Anderson C.D. (2016). Subacute decline in serum lipids precedes the occurrence of primary intracerebral hemorrhage. Neurology.

[50] Kulminski A.M., Raghavachari N., Arbeev K.G., Culminskaya I., Arbeeva L., Wu D., Ukraintseva S.V., Christensen K., Yashin A.I. (2016). Protective role of the apolipoprotein E2 allele in age-related disease traits and survival: Evidence from the Long Life Family Study. Biogerontology.

[51] Rodriguez-Luna D., Rubiera M., Ribo M., Coscojuela P., Pagola J., Piñeiro S., Ibarra B., Meler P., Maisterra O., Romero F. (2011). Serum low-density lipoprotein cholesterol level predicts hematoma growth and clinical outcome after acute intracerebral hemorrhage. Stroke.

[52] Romero J.R., Preis S.R., Beiser A., Decarli C., Viswanathan A., Martinez-Ramirez S., Kase C.S., Wolf P.A., Seshadri S. (2014). Risk factors, stroke prevention treatments, and prevalence of cerebral microbleeds in the framingham heart study. Stroke.

[53] Ding J., Sigurdsson S., Garcia M., Phillips C.L., Eirikdottir G., Gudnason V., van Buchem M.A., Launer L.J. (2015). Risk Factors Associated With Incident Cerebral Microbleeds According to Location in Older People: The Age, Gene/Environment Susceptibility (AGES)-Reykjavik Study. JAMA Neurol..

[54] Charidimou A., Martinez-Ramirez S., Reijmer Y.D., Oliveira-filho J., Lauer A., Roongpiboonsopit D., Frosch M., Vashkevich A., Ayres A., Rosand J. (2017). Total MRI small vessel disease burden in cerebral amyloid angiopathy: A concept validation imaging-pathological study. JAMA Neurol..

[55] Konishi M., Iso H., Komachi Y., Lida M., Shimamoto T., Jacobs D.R., Terao A., Baba S., Sankai T., Ito M. (1993). Associations of serum total cholesterol, different types of stroke, and stenosis distribution of cerebral arteries: The akita pathology study. Stroke.

[56] Lei C., Wu B., Liu M., Chen Y. (2014). Association between statin use and intracerebral hemorrhage: A systematic review and meta-analysis. Eur. J. Neurol..

[57] Song H.B., Jun H.O., Kim J.H., Yu Y.S., Kim K.W., Min B.H., Kim J.H. (2013). Anti-apoptotic effect of clusterin on cisplatin-induced cell death of retinoblastoma cells. Oncol. Rep..

[58] Lehallier B., Essioux L., Gayan J., Alexandridis R., Nikolcheva T., Wyss-Coray T., Britschgi M. (2016). Combined Plasma and Cerebrospinal Fluid Signature for the Prediction of Midterm Progression From Mild Cognitive Impairment to Alzheimer Disease. JAMA Neurol..

[59] Jin J.L., Guo Y.L., Li J.J. (2016). Apoprotein C-III: A review of its clinical implications. Clin. Chim. Acta.

[60] Jensen M.K., Aroner A.A., Mukamal K.J., Furtado J.D., Post W.S., Tsai M.Y., Tjønneland A., Polak J.F., Rimm E.B., Overvad K. (2018). HDL subspecies defined by presence of apolipoprotein C-III and incident coronary heart disease in four cohorts. Circulation.

[61] Morton A.M., Koch M., Mendivil C.O., Furtado J.D., Tjønneland A., Overvad K., Wang L., Jensen M.K., Sacks F.M. (2018). Apolipoproteins E and CIII interact to regulate HDL metabolism and coronary heart disease risk. JCI insight.

[62] Morton A.M., Furtado J.D., Mendivil C.O., Sacks F.M. (2019). Dietary unsaturated fat increases HDL metabolic pathways involving apoE favorable to reverse cholesterol transport. JCI Insight.

[63] Robert J., Button E.B., Martin E.M., McAlary L., Gidden Z., Gilmour M., Boyce G., Caffrey T.M., Agbay A., Clark A. (2020). Cerebrovascular amyloid Angiopathy in bioengineered vessels is reduced by high-density lipoprotein particles enriched in Apolipoprotein E. Mol. Neurodegener..

[64] Koch M., DeKosky S.T., Goodman M., Sun J., Furtado J.D., Fitzpatrick A., Mackey R.H., Cai T., Lopez O.L., Kuller L.H. (2020). High-density lipoprotein and its apolipoprotein-defined subspecies and risk of dementia. J. Lipid Res..

[65] Kinney J.W., Bemiller S.M., Murtishaw A.S., Leisgang A.M., Salazar A.M., Lamb B.T. (2018). Inflammation as a central mechanism in Alzheimer’s disease. Alzheimer’s Dement. Transl. Res. Clin. Interv..

[66] Wojtas A.M., Kang S.S., Olley B.M., Gatherer M., Shinohara M., Lozano P.A., Liu C.-C., Kurti A., Baker K.E., Dickson D.W. (2017). Loss of clusterin shifts amyloid deposition to the cerebrovasculature via disruption of perivascular drainage pathways. Proc. Natl. Acad. Sci. USA.

[67] Miners J.S., Clarke P., Love S. (2017). Clusterin levels are increased in Alzheimer’s disease and influence the regional distribution of Aβ. Brain Pathol..

[68] Hondius D.C., Eigenhuis K.N., Morrema T.H.J., van der Schors R.C., van Nierop P., Bugiani M., Li K.W., Hoozemans J.J.M., Smit A.B., Rozemuller A.J.M. (2018). Proteomics analysis identifies new markers associated with capillary cerebral amyloid angiopathy in Alzheimer’s disease. Acta Neuropathol. Commun..

[69] Camacho J., Moliné T., Bonaterra-Pastra A., Cajal S.R.Y., Martínez-Sáez E., Hernández-Guillamon M. (2019). Brain ApoA-I, ApoJ and ApoE immunodetection in cerebral amyloid angiopathy. Front. Neurol..

[70] Manousopoulou A., Gatherer M., Smith C., Nicoll J.A.R., Woelk C.H., Johnson M., Kalaria R., Attems J., Garbis S.D., Carare R.O. (2017). Systems proteomic analysis reveals that clusterin and tissue inhibitor of metalloproteinases 3 increase in leptomeningeal arteries affected by cerebral amyloid angiopathy. Neuropathol. Appl. Neurobiol..

[71] Schrijvers E.M.C., Koudstaal P.J., Hofman A., Breteler M.M.B. (2011). Plasma clusterin and the risk of Alzheimer disease. JAMA.

[72] Montañola A., de Retana S.F., López-Rueda A., Merino-Zamorano C., Penalba A., Fernández-Álvarez P., Rodríguez-Luna D., Malagelada A., Pujadas F., Montaner J. (2016). ApoA1, ApoJ and ApoE Plasma Levels and Genotype Frequencies in Cerebral Amyloid Angiopathy. Neuromol. Med..

[73] Gupta V.B., Doecke J.D., Hone E., Pedrini S., Laws S.M., Thambisetty M., Bush A.I., Rowe C.C., Villemagne V.L., Ames D. (2016). Plasma apolipoprotein J as a potential biomarker for Alzheimer’s disease: Australian Imaging, Biomarkers and Lifestyle study of aging. Alzheimer’s Dement. Diagn. Assess. Dis. Monit..

[74] Delabar J.M., Ortner M., Simon S., Wijkhuisen A., Feraudet-Tarisse C., Pegon J., Vidal E., Hirschberg Y., Dubois B., Potier M. (2020). Altered age-linked regulation of plasma DYRK1A in elderly cognitive complainers (INSIGHT-preAD study) with high brain amyloid load. Alzheimer’s Dement. Transl. Res. Clin. Interv..

[75] Weinstein G., Beiser A.S., Preis S.R., Courchesne P., Chouraki V., Levy D., Seshadri S. (2016). Plasma clusterin levels and risk of dementia, Alzheimer’s disease, and stroke. Alzheimer’s Dement. Diagn. Assess. Dis. Monit..

[76] Matsubara E., Frangione B., Ghiso J. (1995). Characterization of apolipoprotein J-Alzheimer’s Aβ interaction. J. Biol. Chem..

[77] Yerbury J.J., Poon S., Meehan S., Thompson B., Kumita J.R., Dobson C.M., Wilson M.R. (2007). The extracellular chaperone clusterin influences amyloid formation and toxicity by interacting with prefibrillar structures. FASEB J..

[78] Calero M., Tokuda T., Rostagno A., Kumar A., Zlokovic B., Frangione B., Ghiso J. (1999). Functional and structural properties of lipid-associated apolipoprotein J (clusterin). Biochem. J..

