# Supplementary material for: Comparison of Plasma Lipoprotein Composition and Function in Cerebral Amyloid Angiopathy and Alzheimer’s Disease
Source: Biomedicines. 2021 Jan 12;9(1):72. doi: 10.3390/biomedicines9010072 (PMC7828227; doi:10.3390/biomedicines9010072)
Supplement: Supplementary file 1 [file biomedicines-09-00072-s001.pdf]

biomedicines

MDPI

Supplementary Materials

Table S1. Binary logistic regression for lipid profile and apolipoprotein concentration in plasma.

Parameters	Ctrl – ICH-CAA		Ctrl – AD		ICH-CAA – AD	
	OR (95% CI)	*p* value	OR (95% CI)	*p* value	OR (95% CI)	*p* value
**Chol LDL**	-	-	-	-	1.016 (1.005–1.027)	0.004
**ApoA-II**	0.538 (0.346–0.835)	0.006	-	-	-	-
**ApoE4**	-	-	-	-	2.801 (1.171–6.704)	0.021

Table S2. Binary logistic regression for VLDL composition.

Parameters	Ctrl – ICH-CAA		Ctrl – AD		ICH-CAA – AD	
	OR (95% CI)	*p* value	OR (95% CI)	*p* value	OR (95% CI)	*p* value
**Esterified chol**	0.808 (0.683–0.956)	0.013	-	-	-	-
**ApoC-III**	-	-	-	-	0.39 (0.184–0.829)	0.014
**ApoE4**	-	-	-	-	2.8 (1.184–6.625)	0.019

Table S3. Binary logistic regression for HDL composition.

Parameters	Ctrl – ICH-CAA		Ctrl – AD		ICH-CAA – AD	
	OR (95% CI)	*p* value	OR (95% CI)	*p* value	OR (95% CI)	*p* value
**Esterified chol**	1.583 (1.119–2.239)	0.010	-	-	0.525(0.365–0.754)	0.0005
**ApoC-III**	0.414(0.221–0.772)	0.006	-	-	-	-
**ApoE4**	-	-	-	-	4.529 (1.771–11.579)	0.002

Table S4. Binary logistic regression for ApoJ content in lipoproteins.

Parameters	Ctrl – ICH-CAA		Ctrl – AD		ICH-CAA – AD	
	OR (95% CI)	*p* value	OR (95% CI)	*p* value	OR (95% CI)	*p* value
**ApoJ LDL**	-	-	-	-	1.015 (1.002–1.027)	0.019
**ApoE4**	-	-	-	-	11.092 (1.796–68.5)	0.010

Table S5. Binary logistic regression for lipoprotein size.

Parameters	Ctrl – ICH-CAA		Ctrl – AD		ICH-CAA – AD	
	OR (95% CI)	*p* value	OR (95% CI)	*p* value	OR (95% CI)	*p* value
**LDL size**	-	-	-	-	0.55 (0.331–0.912)	0.021
**Lp-PLA$_2$ activity in HDL (%)**	-	-	-	-	0.962 (0.925–1)	0.049
**ApoE4**	-	-	-	-	3.006 (1.245–7.26)	0.014

A)

	Controls	ICH-CAA	AD	*p*-value
**-/-**	50 (83.3%)	40 (78.4%)	32 (53.3%)	**0.003**
**-/E4**	10 (16.7 %)	9 (17.6 %)	23 (38.3%)	
**E4/E4**	0 (0.0%)	2 (3.9 %)	5 (8.3%)	

	Controls	ICH-CAA	AD	*p*-value
**-/-**	53 (88.3%)	45 (88.2%)	58 (96.7%)	0.342
**-/E2**	6 (10.0 %)	4 (7.8 %)	2 (3.3%)	
**E2/E2**	1 (1.7%)	2 (3.9 %)	0 (0.0%)	

B)

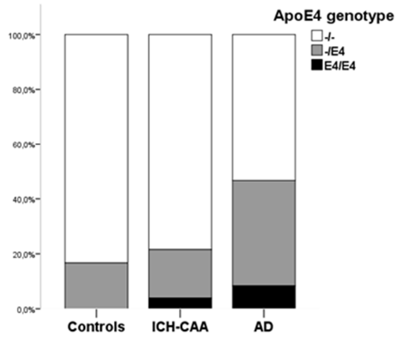

Fgure S1. ApoE genotype. (**A**) Contingency table of ApoE genotype according to clinical diagnosis. (**B**) ApoE4 genotype frequency (%) according to clinical diagnosis.
